# Assessment Methods of Physical Fitness in Wheelchair Tennis Athletes: A Scoping Review and Proposal for a Standard Operating Procedure

**DOI:** 10.3390/jcm14134609

**Published:** 2025-06-29

**Authors:** Ignazio Leale, Alejandro Sánchez-Pay, Valerio Giustino, Michele Roccella, Maria Ruberto, Michele Lattuca, Olga Lo Presti, Manuel Gómez-López, Giuseppe Battaglia

**Affiliations:** 1 Sport and Exercise Sciences Research Unit, Department of Psychology, Educational Science and Human Movement, University of Palermo, 90144 Palermo, Italy; ignazio.leale@unipa.it (I.L.); giuseppe.battaglia@unipa.it (G.B.); 2 Department of Psychology, Educational Science and Human Movement, University of Palermo, 90128 Palermo, Italy; michele.roccella@unipa.it; 3 Department of Physical Activity and Sport, Faculty of Sports Sciences, University of Murcia, Santiago de la Ribera, 30720 Murcia, Spain; aspay@um.es (A.S.-P.); mgomezlop@um.es (M.G.-L.); 4 Department of Education and Sport Sciences, Pegaso Telematic University, 80143 Naples, Italy; maria.ruberto@unipegaso.it; 5 Faculty of Humanities, University of Pegaso, 00187 Rome, Italy; info@koshidobudo.com (M.L.); olga.synda@icloud.com (O.L.P.)

**Keywords:** disability, evaluation, Paralympic Games, standard operating procedure, performance, wheelchair tennis

## Abstract

Wheelchair tennis (WT) is a Paralympic sport designed for athletes with physical impairments. Assessing physical fitness characteristics using appropriate field-based tests and standardized protocols is essential for individualized training, injury prevention, and performance monitoring. However, there is currently limited information on which field-based tests are most suitable and how they should be applied in WT athletes, resulting in inconsistency across studies and practical use. Establishing a standard operating procedure (SOP) enables replicable, cost-effective testing routines that improve data consistency and comparability. We conducted a scoping review to synthesize the existing evidence on field-based physical fitness assessment in WT athletes and to propose a structured SOP for these tests. A comprehensive search was conducted in three electronic databases—NLM PubMed, Web of Science, and Scopus—using predefined keywords and Boolean operators. The inclusion criteria were limited to peer-reviewed, English-language original articles focusing exclusively on field tests in WT athletes. Studies with other populations, reviews, and abstracts were excluded. Eleven studies met the eligibility criteria. This scoping review identified various field tests assessing key fitness components, including cardiorespiratory endurance, muscle strength, agility, and body composition. The most frequently employed tests were the 20 m sprint test, isometric handgrip test, spider test, Illinois Agility Test, and skinfold thickness. These findings highlight the lack of standardized fitness assessments in WT. The proposed SOP offers a practical step toward consistent, replicable, and relevant evaluation in these athletes.

## 1. Introduction

### 1.1. Physical Activity and the Role of Paralympic Sports

Individuals with disabilities generally engage in lower levels of physical activity than those without disabilities [1,2] often due to physical, social, psychological, and financial barriers [3]. This inactivity negatively affects health, leading to reduced aerobic and muscular fitness [4]. In contrast, regular exercise improves health-related fitness [5,6], functional ability [7], postural control, muscle strength, and overall quality of life [8]. It also has positive effects on mood, social behavior, and life satisfaction [9]. 

The Paralympic Games are the highest level of competition in adapted sports, like the Olympic Games. They show the abilities of people with disabilities and promote inclusion, accessibility, and equal opportunities [3,10]. Scientific interest in Paralympic sports has grown over the past decades, focusing on psychological, physiological, biomechanical, and performance-related factors [11]. For example, Leung et al. (2021) demonstrate that sitting volleyball athletes showed greater cardiovascular endurance, improved body composition, and higher physical activity enjoyment than inactive individuals [12]. Similarly, Briley et al. (2023) reported that, despite the physiological fatigue induced by wheelchair rugby match play, athletes were able to maintain sprint performance by modifying their propulsion technique [13]. The media attention on the Paralympics also encourages more people with disabilities to participate in sports and highlights the importance of exercise as a public health prevention strategy [14].

### 1.2. Wheelchair Tennis Characteristics and Demands

Wheelchair Tennis (WT), introduced in 1976, is one of the most popular Paralympic sports [15]. It is an adapted version of conventional tennis that follows the same rules as standing tennis, except that the ball is allowed to bounce twice before being hit. WT has two divisions: the open division, designated for players with a permanent disability affecting one or both lower limbs, and the quad division, for players with an additional permanent upper-limb disability that restricts their ability to handle the racket, execute shots, and maneuver the wheelchair effectively. WT is played on various court surfaces, including hard courts, clay, and grass [16]. In WT, the athletes must develop a comprehensive set of tactical, technical, physical, and psychological skills and biomechanical features [17,18,19]. Warner et al. (2018) identified significant bilateral asymmetries in WT athletes, as well as key differences compared to able-bodied players, underscoring the importance of targeted injury prevention strategies and sport-specific training programs [11]. The physical demands of WT encompass strength [20], power [21], balance [22], coordination [23], agility [23], and aerobic endurance [24]. Performance is also influenced by contextual and individual variables such as [25] the court surface [26] and the player’s ranking [27], age [28], and sex [29]. Effective training programs should simulate real match demands [30], and field-based fitness testing represents a practical tool for evaluating performance [31].

### 1.3. The Need for Standardization and the Aim of the Review

Despite growing interest, research in adapted sports remains fragmented, with a notable lack of standardized fitness assessment protocols in wheelchair sports [32,33]. This gap limits the comparability of findings and the development of evidence-based training and evaluation practices. Previous studies in other Paralympic sports have also highlighted the need for standardizing evaluation protocols to ensure data reliability and comparability [32,33,34,35]. A standard operating procedure (SOP) provides a structured framework with detailed guidelines and steps for implementing protocols [36]. A SOP is a valuable document for supporting the replicability of test sessions and normalizing data, ensuring consistency across assessments [36,37,38]. Furthermore, the feasibility of field tests compared to laboratory tests should be carefully considered during evaluations, as field tests are often more practical, cost-effective, and time-efficient [39]. Their implementation could promote standardization, improve data comparability across studies, and guide evidence-based practice in both research and daily training. 

Physical fitness is evaluated through multiple components, including cardiorespiratory fitness, muscular strength, flexibility, and body composition, following the ACSM definition [40]. 

Based on these considerations, this scoping review aims to examine the field-based tests used to assess physical fitness in WT athletes and to propose a standardized SOP.

## 2. Methods

This scoping review was conducted in accordance with the Preferred Reporting Items for Systematic Reviews and Meta-Analyses for Scoping Reviews (PRISMA-ScR) checklist and explanation [41]. Although the review was not previously registered, a research protocol was developed before the initiation of the study.

### 2.1. Eligibility Criteria

The eligibility criteria were defined using the Population, Intervention, Comparison, Outcomes, and Study design (PICOs) framework, as recommended by the PRISMA-ScR checklist and explanation [41].

The participants included in this review were athletes practicing WT. The review focused exclusively on articles that assessed athletes’ physical fitness through field-based tests. Only studies published in English were included. This criterion was applied to ensure an accurate interpretation of the content. Studies published up to 25 November 2024, were included to ensure the review reflects the most current evidence available. No restrictions were applied regarding study design, allowing the inclusion of observational, descriptive, longitudinal, and randomized trials. However, reviews, meta-analyses, abstracts, citations, conference abstracts, books, letters, editorials, and non-peer-reviewed manuscripts were excluded to ensure the quality and relevance of the included evidence. The Population, Intervention, Comparison, Outcome, and Study design (PICOS) framework was applied to define the eligibility criteria for this review, as detailed in Table 1.

### 2.2. Information Sources

The relevant studies were identified using a comprehensive search of three electronic databases: PubMed (NLM), Web of Science (TS), and Scopus.

A systematic search strategy was applied using a combination of keywords and Boolean operators. The keywords used to formulate the search string were as follows:

Keywords 1: “wheelchair tennis”, “wheelchair sports”.

Keywords 2: “Physical Fitness”, “Sports Physiology”, “Performance Analysis”, “Exercise Physiology”, “Athletic Performance”.

The following search string was used in all three databases:

(“Wheelchair Tennis” AND “Wheelchair Sports”) AND (“Physical Fitness” OR “Sports Physiology” OR “Performance Analysis” OR “Exercise Physiology” OR “Athletic Performance”).

#### 2.2.1. Data Selection and Management

Following the initial database search, all retrieved articles were exported and the duplicates were removed using EndNote X8 (EndNote version 20.2.1; Clarivate Analytics, New York, NY, USA). Subsequently, a screening process was conducted based on the above-mentioned inclusion and exclusion criteria. This screening involved three phases: reviewing the titles, examining the abstracts, and, finally, assessing the full texts of the articles. The steps of this process are illustrated in the PRISMA flow diagram, following the PRISMA-ScR checklist and explanation [41].

#### 2.2.2. Data Collection and Synthesis

A Microsoft Excel spreadsheet (Microsoft Corp; Redmond, WA, USA) was developed to extract the following information from each study: the first author and year of publication; the sample size; the participants’ gender and age (either as a range or as the mean and standard deviation); the field tests used to evaluate health-related physical fitness components, including cardiorespiratory endurance, muscular strength, flexibility, and body composition; and skill-related components, such as speed and agility, postural balance, coordination, power, reaction time, and speed, as classified by Caspersen and colleagues [42].

## 3. Results

**Figure 1:**
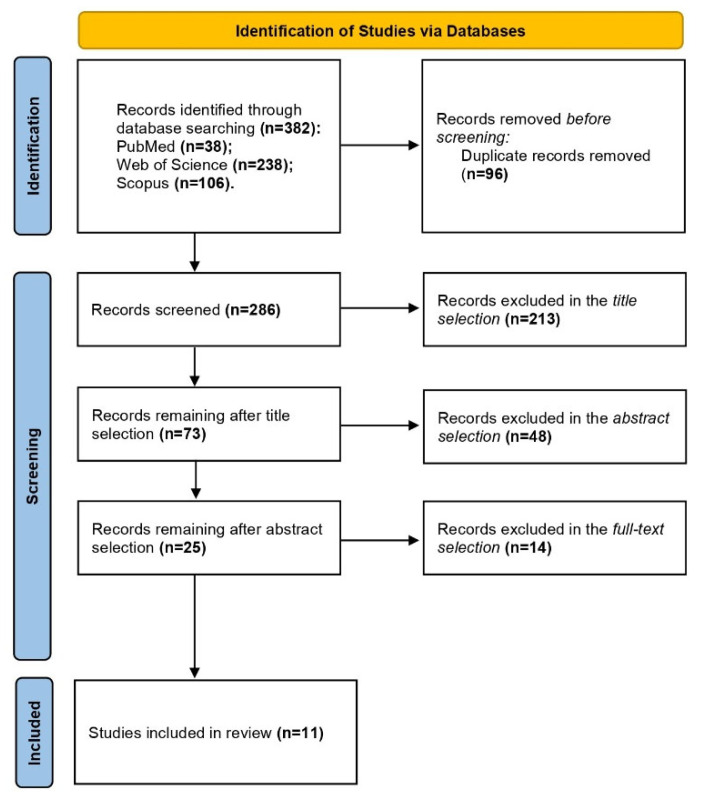
PRISMA flow diagram.

A total of 382 studies were identified through the three databases searched, with 38 studies from PubMed, 106 from Scopus, and 238 from Web of Science. After removing the duplicates, 287 articles remained for eligibility assessment. These were subsequently screened according to the inclusion and exclusion criteria, resulting in 11 studies considered suitable for inclusion in the analysis. A detailed overview of the screening process is provided in the flow diagram (Figure 1). These 11 studies collectively involved 151 wheelchair athletes, of whom 95 were specifically WT athletes. One study did not specify the exact number of WT athletes. All participants were classified as Paralympic, elite, or national-level athletes. Further details about the included studies and their participants are summarized in Table 2.

### 3.1. Characteristics of Included Studies

The current scientific literature includes a limited number of studies that utilize field tests to assess the physical performance of WT athletes [21,28,43,44,45,46,47,48,49,50,51]. These studies primarily focus on four key parameters: cardiorespiratory endurance, muscular strength, speed and agility, and body composition. Cardiorespiratory endurance reflects the ability of the heart and lungs to supply oxygen to the muscles during prolonged exercise [52]. Muscular strength is defined as the capacity of the skeletal muscles to produce measurable force [53]. Speed and agility describe the ability to execute rapid, whole-body movements efficiently [54]. Body composition refers to the proportion of lean body mass and fat mass, which plays a critical role in optimizing performance and mobility [55]. More detailed information, including athlete classification, is presented in Table 2. According to Alannah et al., athletes are categorized as follows: elite athletes compete at the international level; national team athletes participate in national-level competitions; and world-class athletes are those who have won medals at the Olympic Games or World Championships [56].

### 3.2. Cardiorespiratory Endurance

The shuttle run test was the most commonly utilized method to assess cardiorespiratory endurance. This test was adapted from a previously published protocol [57] to meet the specific requirements of the athletes. Vanlandewijck and colleagues [44] developed a version in which the participants repeatedly covered a 25 m distance, marked by two lines, using their wheelchairs. The test started at a speed of 5 km/h, with the velocity increasing by 0.5 km/h every minute. Sensory feedback was provided via a computerized “beep” signal, prompting the participants to cross the target line with at least one wheel. The test concluded when the participant failed to reach the target line on two consecutive attempts, despite verbal encouragement. The total distance covered and the time achieved were recorded. The results indicated that performance on the shuttle run test is significantly influenced by ergonomic and environmental factors, such as surface type and wheelchair characteristics.

De Groot and colleagues [47] proposed an alternative protocol in which the participants repeatedly covered a 20 m distance. The starting speed was also 5 km/h, but the incremental increase was 0.03 km/h per minute. Similar to the previous protocol, the test ended when the participant failed to reach the target line twice consecutively, with both the distance covered and the time recorded as performance outcomes. The findings of this study revealed moderate correlations between VO_2_ peak and performance in the shuttle run test, with shuttle run outcomes also showing a significant association with the athletes’ skill levels.

Another test used to evaluate cardiorespiratory endurance was the Hit and Turn Tennis Test [21], an adaptation of a test originally designed for able-bodied tennis players [58]. In this test, the participants simulated hitting a cone positioned at the intersection of the doubles line and the baseline, synchronized with auditory signals emitted during the test. After each simulated hit, the participants moved to the opposite side to replicate the action, continuing until the sequence concluded. As an incremental test, it ended when the participant could no longer reach the cone at the speed dictated by the auditory signals.

Cardiorespiratory endurance was also evaluated through the multistage fitness test (MFT) [48]. This test required participants to navigate an octagonal path marked by cones within a 15 m × 15 m area. The speed, and consequently the test’s intensity, increased in one-minute stages, with sensory feedback provided via “beep” signals from an audio recording [59]. At each beep, the participants were required to be within the designated turning zone. The test began at a speed of 6 km/h, with increments of 0.37 km/h per stage, and concluded when the participant failed to reach the turning zone three consecutive times. The performance was evaluated based on the number of completed exercise levels (MFT score). The test was employed as an instrument to evaluate the effectiveness of a training program in this population, demonstrating an improvement in aerobic capacity following a circuit training intervention.

Finally, cardiorespiratory endurance was also assessed through a race simulation [45], designed to replicate the physiological demands of an actual match.

### 3.3. Muscle Strength

Muscle strength was assessed in two studies using the isometric handgrip strength test [21,49]. This test was conducted with participants seated in their wheelchairs, elbows extended, and arms positioned such that the dynamometer was parallel to their side without contacting the wheelchair [60,61]. The protocol involved performing two or three maximum isometric contractions, each lasting 3 s [62], with a 120 s rest period between trials. The highest value recorded was used for the analysis. This test is valid and reliable [63,64], making it the ideal tool for strength evaluation. Sánchez-Pay et al. (2023), using this test, reported a progressive increase in muscle fatigue in the dominant hand across consecutive matches, a pattern not observed in the non-dominant hand [49]. These findings highlight the importance of considering hand dominance in recovery strategies and injury prevention protocols for WT athletes [49].

### 3.4. Speed and Agility

The most utilized test to assess speed and agility was the 20 m sprint test (*n* = 5). This test involves three maximal wheelchair sprints, performed either with or without a racket, with a 120 s rest period between each trial. The best performance, recorded in seconds (s) and milliseconds (ms), was used for the analysis. At the start of each sprint, the participants were positioned 0.5 m behind the starting line. In some studies, partial times were also recorded at 5 m and 10 m, providing additional data for a more detailed evaluation of performance.

Other tests utilized included the butterfly sprint agility test [28], which involves completing a designated course in the shortest possible time. This test, adapted for wheelchair athletes, is characterized by sprints combined with directional changes [65]. The agility of the athletes was also assessed using other tests, including the spider test, which was employed in two studies [28,51]. This test required the participants to complete a maneuverability course as quickly as possible, starting from a stationary position with the front wheels behind the start/finish line [66]. The participants propelled their wheelchair forward, performed a slalom course consisting of five cones spaced 1.5 m apart, and then returned to the starting point [66]. The time taken to complete the course was recorded. Rietveld et al. (2024) [51] employed this test to evaluate the effectiveness of a newly designed hand rim for wheelchair tennis players. The findings indicated a reduction in the average rotational speed toward the racket side when using the new rim compared to the standard version. However, the total test completion times remained comparable between the two conditions. These results suggest that while the modified rim may negatively influence rotational movements, it does not significantly impact the overall test performance [51].

The Illinois Agility Test was utilized in two studies [28,51]. This test featured an agility course defined by four cones that marked the test area. Following a sensory stimulus, the athlete ran 9.20 m, turned, and returned to the starting line. The athlete then performed four slaloms around the cones and finished the test with two sprints of 9.20 m. The time taken to complete the test was recorded, and the best performance from the three trials was used for the analysis.

Additional assessments included the turning exercise, where the participants were instructed to propel their wheelchair, execute a 180° turn, and return to the starting point. The best time recorded from three attempts was used for the analysis.

The linear mobility drill was another test employed, involving repeated bouts of acceleration, braking, and backward pulling maneuvers. Performance in this drill was evaluated based on the total time taken to complete the exercise.

Agility was further assessed using the agility *T*-test [21], which has been adapted for wheelchair sports [61]. The test included both accelerations and decelerations, as well as turns in both directions. Participants started from the center of the court, positioned behind the baseline, and were required to move toward the intersection of the single line with the service line, passing through the central area of the court before returning to the starting point. Each participant completed the test three times without a racket and three times with a racket, with a 2 min rest period between each trial. The best result from all trials was recorded for the analysis.

### 3.5. Body Composition

Only one study [43], which involved WT and wheelchair rugby players, investigated the body composition of wheelchair athletes. Specifically, Goosey-Tolfrey and colleagues applied the skinfold test, measuring the thickness of skinfolds at four designated sites [67].

### 3.6. Specific Sports Skills

The serve velocity test [21] was utilized to evaluate specific sports skills, specifically the ability to execute a serve. Each participant performed 10 serves at maximum velocity, and the average velocity of these serves (measured in km/h) was recorded for the analysis. Another specific sports skill was assessed using three medicine ball tests [21] employing a 2 kg medicine ball. These tests simulated forehand, backhand, and serve shots [68,69]. The participants stood behind the throwing line at a 45° angle, with a 15 m measuring tape placed on the court perpendicular to the throwing line. Two evaluators recorded the bounce zone of the thrown ball in 0.10 m increments. Each type of throw was performed three times, with a 2 min rest period between repetitions. Additional information about the field tests conducted in the studies can be found in Table 3. 

## 4. Discussion

This scoping review identified a limited number of studies in the scientific literature that use field tests to evaluate the physical performance of WT athletes. Field tests are valuable as they provide practical, valid, and reliable methods for evaluating athletic performance under sport-specific conditions [70,71]. However, their limited application in the WT context reveals a significant gap in the scientific literature and highlights the need for further research in this area. 

The studies included in this scoping review assessed four key parameters: cardiorespiratory endurance, muscle strength, agility, and body composition. These parameters are essential for understanding the physical and physiological demands of WT, playing a crucial role in optimizing training programs and minimizing the risk of injuries. Cardiorespiratory endurance, commonly measured through shuttle run tests, reflects the efficiency of the cardiovascular and respiratory systems in supplying oxygen during sustained physical effort [52]. Although useful, this test can be influenced by ergonomic and environmental factors such as the surface type and wheelchair setup, potentially limiting their accuracy in this population. Muscle strength is defined as the ability of the skeletal muscles to generate measurable force, torque, or moment around one or more joints. This is typically assessed during a single maximal voluntary contraction under controlled conditions, which include the specificity of the movement pattern, the type of muscle contraction (concentric, isometric, or eccentric), and the contraction velocity [72]. The handgrip test is the most used method to assess muscle strength in WT athletes. However, like other wheelchair sports [73,74], an alternative or complementary method could be the medicine ball throw test. This test evaluates upper body power by measuring the maximum distance an athlete can throw a medicine ball. To ensure consistency and comparability, it is important to standardize the weight of the ball and the number of throws performed during the assessment. Speed and agility are defined as the ability to perform rapid whole-body movements that involve changes in velocity or direction in response to a stimulus [75]. Tests such as the 20 m sprint test, the spider test, and the Illinois Agility Test help quantify these abilities. Agility performance, in particular, is influenced not only by physical traits (strength, power, coordination) but also by cognitive abilities like anticipation and decision-making [75]. Body composition provides valuable insights into an athlete’s overall physical condition, contributing to the balance of lean body mass and body fat levels to enhance performance and mobility [76]. Skinfold measurements are a commonly used and cost-effective method in field settings. Despite the relevance of these performance parameters, this review highlights a notable gap: the lack of specific tests aimed at assessing muscle flexibility, a key factor in injury prevention and range of motion [77,78]. One of the commonly used tests for wheelchair athletes is the back scratch test, which is included in the Brockport Physical Fitness Test manual [79]. This test evaluates the distance between the second fingers of each hand when reaching behind the back: one over the shoulder and the other along the back [80]. A score of 0 is recorded when the fingers touch. The simplicity of this test makes it an effective method for assessing the muscle flexibility of wheelchair athletes. Other tests frequently used in tennis players to measure range of motion include assessments of internal and external shoulder rotation strength [81], which are crucial for both tennis strokes and the biomechanical demands of wheelchair propulsion [49]. This highlights the necessity for future research to develop and validate field tests adapted to the demands of WT athletes.

### 4.1. Strengths and Limitations

This scoping review provides a comprehensive overview of the existing literature on flexibility assessment and shoulder function in WT athletes, an area that has been relatively underexplored. The results of this study could be particularly useful for coaches and sports professionals in developing a standardized battery of tests to assess physical fitness in WT athletes. A SOP could help monitor functional status, guide individualized training, and support injury prevention strategies.

The current body of literature in this area remains limited, making it challenging to generalize findings and establish normative data for WT athletes. In addition, the review did not adopt the Joanna Briggs Institute (JBI) methodology and was not registered on the Open Science Framework (OSF), a factor that may affect its reproducibility. Future reviews may benefit from a more rigorous methodological approach, including protocol registration and adherence to the JBI framework. Addressing these aspects could provide valuable support to coaches and sports scientists, enhancing training programs and enabling more effective monitoring of the development of WT athletes.

### 4.2. Standard Operating Procedure

The following SOP was developed based on a comprehensive review of the existing literature and the evidence synthesized in the present scoping review. It aims to guide research practice, promote methodological standardization, and provide a preliminary framework for future validation. It includes skinfold test sites (biceps, triceps, subscapular, and supra-iliac) for assessing body composition. Alternatively, measurement at two skinfold sites may be used due to the simplicity, cost-effectiveness, and validity as an accurate field assessment method [82,83]. This two-point method also facilitates faster and easier administration compared to the four-point skinfold assessment.

Participants begin with a 10 min warm-up that includes joint mobility exercises, linear wheelchair movements, circular movements, and turns that simulate a hitting action, as well as low-intensity accelerations and decelerations [60]. The test battery is structured to evaluate various physical performance components. Muscle strength is assessed using the isometric handgrip strength test, while flexibility is evaluated with the back scratch test or by measuring internal and external shoulder rotation. Speed and agility are analyzed using the 20 m sprint test, the spider test, and the Illinois Agility test. Finally, cardiorespiratory endurance is measured with the shuttle run test. The participants should be instructed to perform all tests at maximum intensity, with sufficient rest between trials, and they are allowed to familiarize themselves with the procedures. To ensure consistency, each participant should perform the tests using their tennis wheelchair. The detailed SOP is presented in Table 4.

The proposed SOP represents an initial step, developed through the analysis of published articles and conclusions drawn from the results of previous studies. The next step should focus on assessing the validity and feasibility of the SOP through original research.

### 4.3. Practical Implications

The development of a SOP for the physical fitness evaluation of WT athletes will significantly contribute to advancing this field on a global scale. It will facilitate the comparison of findings across studies and support the creation of normative data based on players’ ability levels and experience. Furthermore, the SOP will aid coaches in analyzing team data and understanding their athletes’ fitness levels, ultimately enabling the design of more effective training programs.

## 5. Conclusions

In conclusion, this scoping review shows the limited number of studies employing field-based tests to evaluate physical fitness in WT athletes. The lack of standardized methods limits the comparability of the results and limits the development of evidence-based training protocols. To address this gap, we propose a SOP that offers a practical, replicable approach for evaluating WT athletes’ fitness. This SOP may support coaches and researchers in monitoring performance and optimizing individualized training. However, further studies are needed to validate and refine this procedure to ensure its reliability, feasibility, and applicability across various competitive levels.

## Figures and Tables

**Table 1:** PICOS framework.

PICOS Framework	Details
Population	Athletes practicing wheelchair tennis
Intervention	Field-based physical fitness assessments or tests conducted on wheelchair tennis athletes
Comparator	Not applicable
Outcomes	Measures of physical fitness components such as cardiorespiratory fitness, muscular strength, flexibility, and body composition
Study design	Peer-reviewed original research articles reporting on field testing or physical fitness assessments of wheelchair tennis athletes Exclusion of reviews, editorials, and conference abstracts

**Table 2:** General information related to the studies (*n* = 11) included in the review.

Reference	Ntot [Nwt] [m] [f]	Age [Years ± SD]	Level	Physical Fitness
Goosey-Tolfrey et al., 2006 [43]	8 [4] [8] [0]	29 ± 4.0	NT	Cardiorespiratory endurance
Vanlandewijck et al., 2006 [44]	11 [Na] [11] [0]	31 ± 6.62	Elite	Cardiorespiratory endurance—speed and agility
Bernardi et al., 2010 [45]	34 [4] [34] [0]	33.1 ± 7.75	PA	Cardiorespiratory endurance
Mason et al., 2011 [46]	14 [3] [11] [3]	23 ± 6	Elite	Speed and agility
De Groot et al., 2016 [47]	15 [15] [15] [0]	21.2 ± 8.4	Elite	Cardiorespiratory endurance
Rietveld et al., 2019 [28]	21 [21] [9] [12]	27.3 ± 8.7 (A) 14.8 ± 1.5 (J)	Elite	Speed and agility
Sánchez-Pay et al., 2021 [21]	9 [9] [9] [0]	38.4 ± 11.28	NT	Cardiorespiratory endurance–muscle strength–speed and agility–specific sports skills
Yulianto et al., 2021 [48]	14 [14] [10] [4]	31–35 *	NT	Cardiorespiratory endurance
Sánchez-Pay et al., 2023 [49]	8 [8] [8] [0]	38 ± 10	NT	Muscle strength
Rietveld et al., 2024 [50]	8 [8] [6] [2]	34 ± 9	Elite	Speed and agility
Rietveld et al., 2024 [51]	9 [9] [5] [4]	27 ± 15	Elite	Speed and agility

(**Nwt**), number wheelchair tennis; (**m**), male; (**f**), female; **SD**, standard deviation; (**NT**), national team; (**Na**)**,** not applicable; (**PA**), Paralympic athletes; (**A**)**,** adults; (**J**)**,** juniors; (*), interval.

**Table 3:** Information on the field tests included in the studies.

Reference	Cardiorespiratory Endurance	Muscle Strength	Speed and Agility	Body Composition	Specific Sports Skills	Main Findings
Goosey-Tolfrey et al., 2006 [43]				ST		Body composition was assessed through the sum of four ST, with values indicating moderate variability among athletes.
Vanlandewijck et al., 2006 [44]	SR		TE			SR times differed significantly across conditions; VO_2_peak similar; SR performance explained up to 41% of VO_2_peak variance.
Bernardi et al., 2010 [45]	RS					VO_2_peak and ventilatory threshold were significantly higher in Nordic sit skiing and wheelchair racing athletes compared to WT; strong correlation between field test VO_2_ and lab-measured aerobic fitness.
Mason et al., 2011 [46]			20 m sprint; LMD			18° camber angle improved 20 m sprint times, acceleration, and linear mobility compared to 24°, with large effect sizes; 24° camber impaired performance, likely due to increased drag.
De Groot et al., 2016 [47]	SR					SR strongly correlated with player skill level, but poorly predicted VO_2_peak.
Rietveld et al., 2019 [28]			5,10,20 m sprint; BS; ST; IAT			Four WT field tests showed good construct validity, effectively distinguishing talented juniors from international adults.
Sánchez-Pay et al., 2021 [21]	HTTT	Handgrip	5,10,20 m sprint; *T*-test;	SVT; MBT		MBT was the strongest predictor of serve velocity in male WT players, showing high correlations with serve and stroke speeds.
Yulianto et al., 2021 [48]	MFT					Circuit training program, measured with MFT, showed good content validity and significantly improved aerobic endurance in WT athletes after 6 weeks.
Sánchez-Pay et al., 2023 [49]		Handgrip				Successive WT matches caused a significant decrease in dominant handgrip strength, with strength declining from first to fourth match.
Rietveld et al., 2024 [50]			10 m sprint			Higher tire pressure reduced drag; power loss varied by surface (hard court < clay < grass).
Rietveld et al., 2024 [51]			20 m sprint; ST; IAT			New hand rim showed slight improvements in work per push, peak velocity, and distance during lab sprint tests; no significant differences found in field tests.

(**ST**), skinfold thicknesses; (**SR**), shuttle run; (**TE**), turning exercise; (**RS**), race simulation; (**LMD**), linear mobility drill; (**BS**), butterfly sprint; (**ST**), spider test; (**IAT**), Illinois Agility Test; (**HTTT**), Hit and Turn Tennis Test; (**SVT**), serve velocity test; (**MBT**), medicine ball tests; (**MFT**), multistage fitness test.

**Table 4:** Standard operating procedure for the evaluation of the physical fitness of wheelchair tennis athletes.

Physical Fitness Component	Test Adopted
Body composition	Two-site skinfold thickness
**Warm-up**	
Muscle strength	Isometric handgrip strength test
Flexibility	Back scratch test/shoulder rotation
Speed and agility	20 m sprint/spider test/Illinois Agility Test
Cardiorespiratory endurance	Shuttle run test
